# Trigocherrierin A, a Potent Inhibitor of Chikungunya Virus Replication

**DOI:** 10.3390/molecules19033617

**Published:** 2014-03-24

**Authors:** Mélanie Bourjot, Pieter Leyssen, Johan Neyts, Vincent Dumontet, Marc Litaudon

**Affiliations:** 1 EA4267 Epithelial Functions and Dysfunctions, UFR of Medical and Pharmaceutical Sciences, 19 rue Ambroise Paré, 25030 Besançon, France; E-Mail: melanie.bourjot@univ-fcomte.fr; 2 Rega Institute for Medical Research (KU Leuven), Minderbroedersstraat 10, B3000, Leuven, Belgium; E-Mails: Pieter.Leyssen@rega.kuleuven.be (P.L.); Johan.Neyts@rega.kuleuven.be (J.N.); 3 Gif Research Center, Institute of Chemistry of Natural Substances (ICSN), CNRS, Labex CEBA, 1, avenue de la Terrasse, 91198 Gif sur Yvette Cedex, France; E-Mail: vincent.dumontet@cnrs.fr

**Keywords:** *Trigonostemon cherrieri*, Euphorbiaceae, chikungunya virus (CHIKV), daphnane diterpenoid orthoester (DDO)

## Abstract

Trigocherrierin A (**1**) and trigocherriolide E (**2**), two new daphnane diterpenoid orthoesters (DDOs), and six chlorinated analogues, trigocherrins A, B, F and trigocherriolides A–C, were isolated from the leaves of *Trigonostemon cherrieri*. Their structures were identified by mass spectrometry, extensive one- and two-dimensional NMR spectroscopy and through comparison with data reported in the literature. These compounds are potent and selective inhibitors of chikungunya virus (CHIKV) replication. Among the DDOs isolated, compound **1** exhibited the strongest anti-CHIKV activity (EC_50_ = 0.6 ± 0.1 µM, SI = 71.7).

## 1. Introduction

Chikungunya is an acute illness that is characterized by fever, rash and arthralgia. The chikungunya virus (CHIKV) that causes this disease is an alphavirus that belongs to the Togaviridae family [1], transmitted by different mosquito species, including the Asian tiger mosquito (*Aedes albopictus*, Culicidae), one of the most invasive in the World. In the past decade, CHIKV has re-emerged in Africa, Asia and in the Indian Ocean islands, and during these outbreaks was associated with a high impact and severe morbidity. Due to climate changes and the ability of *A. albopictus* to now survive in more temperate areas, this disease has also become a worldwide threat [2]. Recently, the first outbreaks have been reported in the Americas [3,4]. Currently, no specific antiviral therapy or a vaccine is available for the treatment or prevention of this disease.

In an effort to identify novel inhibitors of CHIKV replication, we selected the rare endemic New Caledonian species *Trigonostemon cherrieri* for a thorough chemical investigation. Phytochemical investigations of *Trigonostemon* species began in the 90s and have dramatically increased during the last five years. Phenanthrenes [5,6] alkaloids [7,8], various daphnane and tigliane-type diterpenoids [9,10,11] were isolated from various species of this genus, many of the latter being known to possess antiviral properties [12,13,14,15]. From the bark and wood of *T. cherrieri*, we recently reported the isolation and structural characterization of trigocherrins A-F and trigocherriolides A–D, unusual chlorinated daphnane diterpenoid orthoesters (DDO) [16,17]. These results prompted us to make the complete chemical investigation of the leaves of this species. As a result, in this paper we report the isolation, characterization and anti-CHIKV activities of two new analogues, trigocherrierin A (**1**) and trigocherriolide E (**2**), along with trigocherrins A, B and F, and trigocherriolides A, B and C, from the leaves of *T. cherrieri*. Trigocherrierin A (**1**) is the only analogue of this chemical series free of chlorine atoms in its structure.

## 2. Results and Discussion

**Figure 1:**
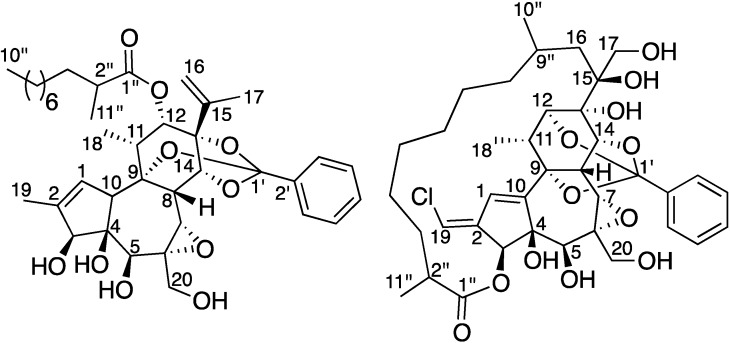
Structures of trigocherrierin A (**1**) and trigocheriolide E (**2**).

The air-dried powder of the leaves of *T. cherrieri* was extracted with EtOAc to give a crude extract, which was partitioned between hexane and aqueous MeOH. The aq. MeOH fraction was then subjected to LH-20 liquid chromatography. The active fractions (F5, F6 and F7) were then repeatedly purified by LH-20, preparative and semi-preparative C_18_ HPLC to yield trigocherrins A, B, F, trigocherriolides A, B, C, and E (**2**), and trigocherrierin A (**1**) in trace quantities (Figure 1).

**Figure 2:**
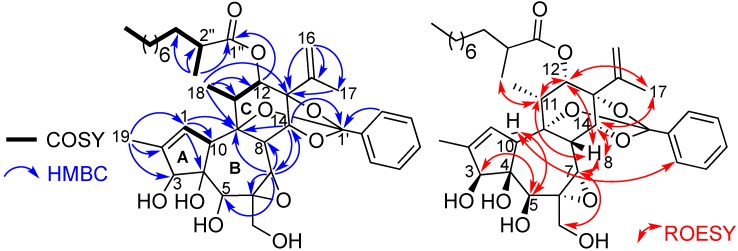
Key HMBC and COSY (left), and ROESY (right) correlations of compound **1**.

Trigocherrierin A (**1**) possesses the molecular formula C_38_H_52_O_10_, based on its protonated molecular ion peak at *m*/*z* 669.3652 [M+H]^+^, obtained by HR-ESIMS (calcd. 669.3639), thus requiring 13 degrees of unsaturation. In accordance with the molecular formula, the ^13^C-NMR data in combination with analysis of the HSQC spectrum revealed 38 carbons signals due to five methyls, nine methylenes (one olefinic), 15 methines (five oxygenated and six olefinic), and nine quaternary carbons (one ester carbonyl, five oxygenated and three olefinic). The 1D and 2D NMR spectra revealed signals attributable to a daphnane diterpenoid orthoester and showed the presence of an isopropenyl group [*δ*_C_ 142.5, 113.2 and 19.8 (C-15, C-16 and C-17, respectively) and *δ*_H_ 4.98 and 5.17 (H_2_-16), 1.73 (H_3_-17)], a benzene ring (*δ*_H_ 7.3-7.8/*δ*_C_ 126.4-135.7), and an aliphatic side chain at *δ*_H_ 0.85 (H_3_-10'')/14.3 (C-10''), 1.12-1.26 (H_2_-4'' to H_2_-9'')/22.9-32.1 (C-4'' to C-9''), 1.64 (H-3'')/33.6 (C-3''), 2.44 (H-2'')/40.0 (C-2''). The COSY correlation between H-1 and H-10, associated with HMBC correlations from H-1 to C-4, C-9, C-10, and from H_3_-19 to C-1, C-2 and C-3, allowed to build ring A. The construction of rings B and C, and the junctions A/B and B/C were deduced from COSY and HMBC correlations as depicted in Figure 2. The presence of a trisubstitued epoxide at positions 6 and 7 on ring B, was suggested from the molecular formula, the chemical shifts of C-6 and C-7 at *δ*_C_ 61.5 and 63.9, respectively, and HMBC correlations from H-7 to C-6, C-8, C-9 and C-14. The observation of a large ^1^*J*_C-H_ coupling constant value of 170 Hz for H-7/C-7 confirmed the presence of this epoxide. From HMBC correlations H_3_-18/C-9/C-11/C-12, and H_2_-16/H_3_-17/C-13/C-15, it can be deduced the locations of the secondary methyl and isopropenyl groups at C-11 and C-13, respectively, as depicted in Figure 2. The position of the secondary CH_3_-11'' group at C-2'', and the attachment of the aliphatic side-chain at C-12, via an ester linkage, were supported by HMBC correlations from CH_3_-11'' to C-1''/C-2'' and C-3'', and from H-12 to C-1'', respectively. The quaternary carbon at *δ*_C_ 118.3 is characteristic of a 9,13,14-orthobenzoate moiety [18]. The presence of the latter was confirmed by HMBC correlations from H-14 to C-1'/C-9 and C-7.

**Figure 3:**
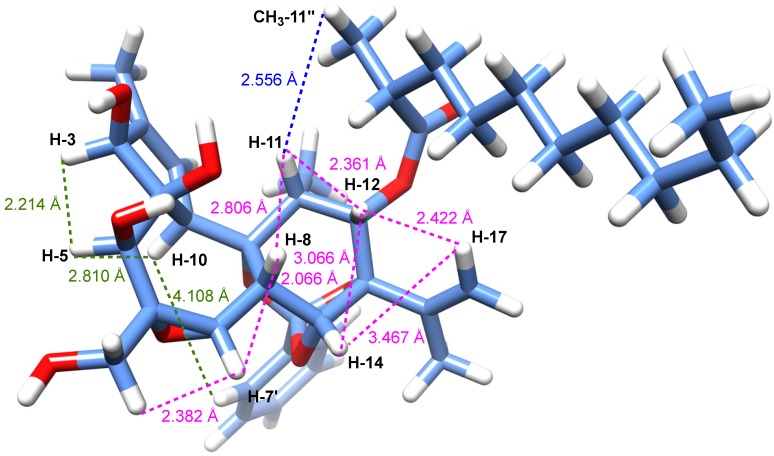
3D representation of a possible conformer of **1** as derived from energy minimization showing distances (Å) between ROE-interacting protons (distances are shown in dotted lines in green and magenta for protons below and above the plan, respectively). The R* configuration was assigned arbitrarily for C-2''.

The relative configuration of compound 1, with the exception of the stereocenter C-2'', could be determined, thanks to analysis of ROESY correlations and after an energy minimization study (Figure 2 and Figure 3). Cross peaks observed between protons H-11/H-12, H-12/H-8, H-8/H-7, H-8/H-11, H-8/H-14, H-14/H-17, H-17/H-12 indicated that they all had the same orientation that we arbitrary fixed as *β*. A typical vicinal coupling constant value of 7.6 Hz between H-11 and H-12 confirmed that the aliphatic side chain at C-12 is *α*-oriented, otherwise the value would be 0 [12,19]. Other ROESY correlations were observed between H-3 and H-5, H-5 and H-10, and H-10 and H-3' (or H-7'), indicating that they all are on the *α*-face of the molecule as depicted in Figure 2. The latter, although weak, is essential because it allowed us to determine the relative configuration of all stereogenic centers of the tricyclic core as shown in Figure 2. However, to ascertain the *β*-orientation of C-3, C-4 and C-5 hydroxyl groups and *α*-orientation of H-10 in compound 1, the structure was subjected to energy minimization with respect to all atoms by using Avogadro 1.1.1 software (MMFF94(s) force field, algorithm Steepest Descent). The protons interatomic distances were measured and the most relevant distances are shown in Figure 3. The results of this study indicated clearly that all ROESY correlations were in agreement with the proton interatomic distances measured on the energy-minimized structure (Figure 3). In particular, it can be observed a spatial proximity between protons H-3, H-5, H-10 and H-7' on one hand, and H-8, H-11, H-12, H-14 and H-17 on the other hand, corroborating the structural study. Several DDOs isolated from *T. thyrsoideum*, such as trigonosins A and B [18] and trigonothyrine F and G [12], possess similar carbon skeleton substituted by a 9,13,14-orthobenzoate moiety and various hydroxy and acetoxy groups. For these compounds, it is interesting to note that hydroxy or acetoxy groups at C-3, C-4 and C-5 are *β*-oriented and proton H-10 *α*-oriented, as it was the case for all compounds of the trigocherrin and trigocherriolide chemical series [16,17]. Unlike the other members of the trigocherrins and trigocherriolides chemical series, trigocherrierin A is the only one lacking of a chlorine atom and having a 9,13,14-orthobenzoate moiety.

**Figure 4:**
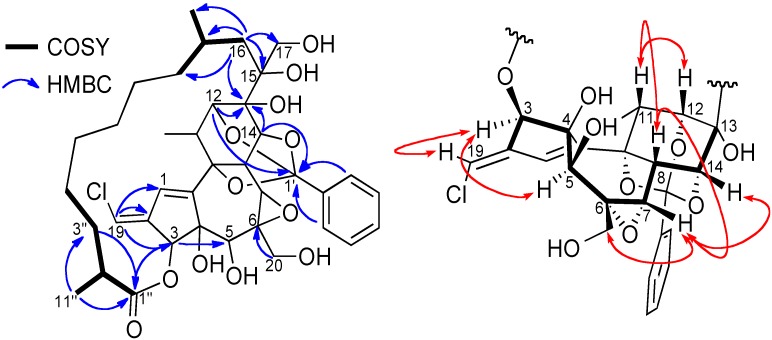
.Key COSY and HMBC (left), and ROESY (right) correlations of compound **2**.

Compound **2** possesses the molecular formula C_38_H_49_O_12_Cl, based on its quasi-molecular ion peak at *m*/*z* 733.3018 [M+H]^+^ obtained by HR-ESIMS (calcd. 733.2991), thus requiring 14 degrees of unsaturation. The 3:1 ratio of [M+H]^+^ and [M+2+H]^+^ obtained by ESIMS indicated that **2** possesses one chlorine atom. Its IR spectrum showed characteristics absorption bands at 3,460 cm^−1^ for hydroxyl groups and 1,710 cm^−1^ for an ester carbonyl group. The chemical shifts and multiplicities of the ^1^H and ^13^C-NMR signals of compound **2** were closely related to those of trigocherriolides B and C [16], suggesting that compound **2** has a macrocyclic DDO backbone bearing one monosubstituted aromatic ring and a vinyl chloride moiety. The latter was confirmed by the high value of the ^1^*J*_C-H_ coupling constant (195 Hz) observed for H-19/C-19 on the HMBC spectrum [20]. The HSQC spectrum revealed the presence of three methyls, nine methylenes (two oxymethylenes), 16 methines (five oxygenated and seven olefinic) and ten quaternary carbons (one ester carbonyl, six oxygenated and three olefinic). In the HMBC spectrum, cross peaks from H-19 to C-1, C-2 and C-3 confirmed the position of the vinyl chloride on the five-membered ring A. The position of the orthobenzoate moiety at C-9, C-12, and C-14 is suggested by the typical chemical shift of the quaternary carbon C-1' at *δ*_C_ 108.8 [21]. This location was confirmed by HMBC correlations from H-3', H-7', H-12 and H-14 to C-1'. An eleven carbons aliphatic side chain attached at the carbonyl ester C1'' on one side and at the quaternary carbon C-15 on the other side can be constructed with the help of ^1^H-^1^H COSY and HMBC experiments (Figure 4), and by deduction from the molecular formula. Indeed, in the HMBC spectrum, cross peaks from H-3 (*δ*_H_ 5.20), H_2_-3'' (*δ*_H_ 1.35 and 1.64) and Me-11'' (*δ*_H_ 1.15) to carbonyl C-1'' (*δ*_C_ 178.1) indicated the esterification of the daphnane skeleton at position 3 by an aliphatic substituent, whereas the second anchor point of the aliphatic side chain to the daphnane core at C-13 via the oxy-quaternary carbon C-15 is supported by correlations from H-16 (*δ*_H_ 1.63) to C-13, C-15 C-17, C-8'', C-9'' and C-10'', and from H-12 and H-14 to C-13. The location of the second oxymethylene groups at C-6 was established thanks to HMBC correlation from H-20 (*δ*_H_ 3.96) to C-6.

The relative stereochemistry of compound **2** was determined by a careful analysis of its ROESY spectrum and through comparison with ^1^H and ^13^C-NMR data of that of trigocherriolides A-D [16]. Cross peaks between H-12/H-11, H-11/H-8, H-8/H-7 and H_2_-20/H-7/H-14 indicated that these protons have the same orientation, arbitrarily fixed as *β*, whereas the H-3/H-5 cross peak suggested a *β*-orientation of the ester aliphatic side chain at C-3 and the hydroxyl group at C-5 as depicted in Figure 4.

Finally, the cross peak between the vinylic proton H-19 and H-3 indicated the stereochemistry of the double bond as *E*. The relative stereochemistry of the macrolactone was not determined due to its high flexibility and the long distance between stereogenic centers C-2'' and C-9'' with other ones. All these data allowed us to propose the structure depicted in Figure 1 for trigocherriolide E (**2**).

The antiviral potency of compounds **1** and **2** was evaluated in a virus-cell-based assay against CHIKV. Compounds **1** and **2** reproducibly inhibited CHIKV-induced cell death with EC_50_ of 0.6 ± 0.1 and 0.7 ± 0.1 µM (n = 3), respectively, and only caused a significant anti-metabolic effect at a concentration of 43 ± 16, and 6.6 ± 0.6 µM (CC_50_), allowing to calculate a selectivity index (SI or window for antiviral selectivity calculated as CC_50_ Vero/EC_50_ CHIKV) of 71.7 and 9.4, respectively. When compared with the biological data that were previously reported for trigocherrins A, B and F, and trigocherriolides A-C [16,17], trigocherrierin A (1) exhibited the strongest anti-CHIKV activity as is apparent from its lower EC_50_ and higher SI values. From these results, it can be deduced that the chlorine atom is not essential for the anti-CHIKV activity, and that a different location of the orthobenzoate moiety at C-9, C-13, and C-14 (instead of C-9, C-12, and C-14 for other compounds of the series), does not affect the antiviral activity or selectivity. The anti-CHIKV activity of trigocherriolide E (**2**) is similar to that of trigocherriolides A–C, but with a slightly lower anti-metabolic effect (or more pronounced adverse effect on the host cells).

## 3. Experimental

### 3.1. General Information

Optical rotations were determined at 25 °C with a JASCO P1010 polarimeter. UV spectra were recorded using a Perkin-Elmer Lamba 5 spectrophotometer. IR spectra were performed on a Nicolet FT-IR 205 spectrophotometer. NMR spectra were recorded in CDCl_3_ on a Bruker Avance 600 MHz instrument with TMS as internal standard, using a 1.7 mm microprobe. HR-ESIMS data were acquired on a Thermoquest TLM LCQ Deca ion-trap spectrometer. Silica gel (6–35 µm) and analytical plates (Si gel 60F 254) were purchased from SDS (Val de Reuil, France). Sephadex LH-20 was purchased from Sigma-Aldrich (Lyon, France). Kromasil analytical, semipreparative, and preparative C_18_ columns (250 × 4.5, 250 × 10, and 250 × 21.2 mm; i.d. 5 µm, Thermo) were used for HPLC separations using a Dionex autopurification system equipped with a binary pump (P580), a UV-Vis array detector (200–600 nm, Dionex UVD340U), and a PL-ELS 1000 ELSD detector (Polymer Laboratory now part of Varian, Les Ulis, France). All other solvents were purchased from SDS (France).

### 3.2. Plant Material

Leaves of *T. cherrieri* were collected in May 2009 in Poya Region on the west coast of New Caledonia. A voucher specimen (POU-0324) was deposited at the Herbarium of the Botanical and Tropical Ecology Department of the IRD Center, Nouméa, New Caledonia.

### 3.3. Extraction and Isolation

The leaves (1.2 kg) were successively extracted with EtOAc (4 × 1.5 L) and MeOH (4 × 1.5 L) at room temperature. The EtOAc extract (46 g) was subjected to a liquid/liquid partition between *n*-hexanes/MeOH_aq_ (MeOH:H_2_O 90:10) leading to a non-polar fraction (40 g) and a polar fraction (6 g). The polar extract (6 g) was subjected to LH-20 column chromatography using an isocratic of MeOH 100%, leading to 10 fractions F1 to F10. Fraction F4 (925 mg) was subjected to LH-20 column chromatography using an isocratic of MeOH 100%, leading to 10 sub-fractions F4-1 to F4-10. Sub-fraction F4-6 (221.8 mg) was purified onto a preparative C_18_ column using a gradient H_2_O-ACN (40:60 to 100:0 in 25 min) at 21 mL/min to afford trigocherrierin A (1, 0.6 mg). The purification of the sub-fraction F4-7 (98 mg) by semi-preparative HPLC with a C_18_ column using H_2_O-ACN (30:70 to 100:0 in 50 min) at 3 mL/min allowed the isolation of trigocherrin F (0.9 mg), trigocherriolides B (1.4 mg) and C (0.4 mg). Sub-fraction F4-8 (7.8 mg) was purified to a semi-preparative C_18_ column using a gradient (H_2_O-ACN, 30:70 to 100:0 in 50 min at 3 mL/min) to afford trigocherrin F (0.1 mg), trigocherriolides A (0.2 mg), B (0.1 mg), C (0.4 mg) and E (**2**, 0.1 mg). Fraction F6 (365 mg) was subjected to LH-20 column chromatography using an isocratic of MeOH 100%, leading to 9 sub-fractions F6-1 to F6-9. The purification of the sub-fraction F6-5 (60 mg) by semi-preparative HPLC using a gradient H_2_O-ACN (25:75 to 10:90 in 40 min) at 3 mL/min allowed the isolation of trigocherrierin A (1, 0.6 mg) and trigocherrin A (0.3 mg). Sub-fraction F6-6 (56 mg) was purified to a semi-preparative C_18_ column using a gradient H_2_O-ACN (20:80 to 0:100 in 50 min) at 3 mL/min to afford trigocherrin F (0.1 mg), trigocherriolides B (0.8 mg) and C (0.7 mg). The purification of the sub-fraction F6-7 (22 mg) by semi-preparative HPLC with a C_18_ column using H_2_O-ACN (30:70 to 100:0 in 50 min) at 3 mL/min allowed the isolation of trigocherrin B (0.6 mg) and trigocherriolides B (0.7 mg), C (1.2 mg) and E (**2**, 1.0 mg).

### 3.4. Spectral Data

*Trigocherrierin A* (**1**). White amorphous powder; [*α*]^25^_D_ +20 [*c* 0.02, MeOH]; UV [MeOH] *λ*_max_ (log *ε*) 208 (3.92) nm; ^1^H-NMR (CDCl_3_, 600 MHz) and ^13^C-NMR (CDCl_3_, 150 MHz), see Table 1; HRESIMS *m*/*z* 669.3652 [M+H]^+^ (calcd for C_38_H_53_O_10_, 669.3639).

**Table 1:** NMR spectroscopic data (150 and 600 MHz, CDCl_3_) for **1** and **2**.

Position	1		2	
	δ_C_	δ_H_, mult. (*J* in Hz)	δ_C_	δ_H_, mult. (*J* in Hz)
1	127.0	5.64, s	126.2	6.45, s
2	137.0	-	139.9	-
3	83.2	4.36, brs	78.8	5.20, s
4	78.7	-	84.0	-
5	75.5	4.06, s	72.2	4.01, s
6	61.5	-	61.0	-
7	63.9	3.40, s	63.2	3.29, brs
8	35.6	3.21, s	35.0	4.46, brs
9	82.0	-	75.0	-
10	52.2	3.56, s	148.9	-
11	39.2	2.92, q (7.0)	34.9	2.64, m
12	71.5	5.25, d (8.0)	79.5	4.23, brs
13	87.0	-	75.8	-
14	82.6	4.59, brs	79.7	4.55, brs
15	142.5	-	75.8	-
16	113.2	4.98, s/5.17, s	35.9	1.54, m1.63, brd (13.6)
17	19.8	1.73, brs	65.7	3.73, d (10.8)3.83, d (10.8)
18	11.5	1.09, d (7.0)	14.0	1.21, d (7.4)
19	13.8	1.69, s	115.3	6.08, s
20	65.7	3.68, m/3.92, d (11.5)	65.8	3.61, m/3.96, m
1'	118.3	-	108.8	-
2'	135.7	-	138.6	-
3', 7'	126.4	7.73, m	125.4	7.70, m
4', 6'	128.2	7.35, m	128.4	7.38, m
5'	129.7	7.35, m	129.8	7.37, m
1''	176.6	-	178.1	-
2''	40.0	2.44, q (7.0)	41.7	2.46, m
3''	33.6	1.64, m	35.0	1.35, m/1.64, m
4''	27.6	1.12–1.23, m	31.2	1.09, m/1.32, m
5''	29.9	1.12–1.23, m	26.9	1.24, m
6''	29.7	1.12–1.23, m	29.1	1.15, m/1.35, m
7''	29.7	1.12–1.23, m	27.5	1.20, m/1.41, m
8''	32.1	1.12–1.23, m	38.4	1.21, m/1.34, m
9''	22.9	1.26, m	25.9	1.59, m
10''	14.3	0.85, t (7.0)	24.3	0.99, d (6.0)
11''	17.5	1.12, d (7.0)	18.7	1.15, d (7.0)

*Trigocherriolide E* (**2**). White amorphous powder; [*α*]^25^_D_ −47 [*c* 0.1, MeOH]; UV [MeOH] *λ*_max_ (log *ε*) 255 (4.25) nm; IR *υ*_max_ 3460, 1710 cm^−1^; ^1^H-NMR (CDCl_3_, 600 MHz) and ^13^C-NMR (CDCl_3_, 150 MHz), see Table 1; HRESIMS *m*/*z* 733.3018 [M+H]^+^ (calcd for C_38_H_50_O_12_Cl, 733.2991).

### 3.5. Chikungunya Virus-Cell Based Antiviral Assay

Serial dilutions of the plant extract, fractions, or pure substances, as well as of the reference compound chloroquine, were prepared in assay medium [MEM Rega3 (cat. No. 19993013; Invitrogen), 2% FCS (Integro, Zaandam, The Netherlands), 5 mL of 200 mM l-glutamine, and 5 mL of 7.5% sodium bicarbonate] that was added to empty wells of a 96-well microtiter plate (Falcon, BD, Haasrode, Belgium). Subsequently, 50 µL of a 4× virus dilution in assay medium was added, followed by 50 µL of a cell suspension. This suspension, with a cell density of 25,000 cells/50 µL, was prepared from a Vero cell line subcultured in cell growth medium (MEM Rega3 supplemented with 10% FCS, 5 mL of l-glutamine, and 5 mL of sodium bicarbonate) at a ratio of 1:4 and grown for 7 days in 150 cm² tissue culture flasks (Techno Plastic Products Menen, Belgium). The assay plates were returned to the incubator for 6–7 days (37 °C, 5% CO_2_, 95%–99% relative humidity), a time at which maximal virus-induced cell death or cytopathic effect (CPE) is observed in untreated, infected controls.

Subsequently, the assay medium was aspired, replaced with 75 µL of a 5% MTS (Promega, Leiden, The Netherlands) solution in phenol red-free medium, and incubated for 1.5 h. Absorbance was measured at a wavelength of 498 nm (Safire2, Tecan, Mechelen, Belgium); optical densities (OD values) reached 0.6–0.8 for the untreated, uninfected controls. Raw data were converted to percentage of controls, and the EC_50_ (50% effective concentration, or concentration that is calculated to inhibit virus-induced cell death by 50%) and CC_50_ (50% anti-metabolic concentration, or concentration that is calculated to inhibit the overall cell metabolism by 50%) were derived from the dose-response curves. Selectivity Index (SI) was determined as the ratio of CC_50_ to EC_50_. All assay conditions producing an antiviral effect that exceeded 50% were checked microscopically for minor signs of CPE or adverse effects on the host cell (*i.e*., altered cell morphology, *etc*…). A compound is only considered to elicit a selective antiviral effect on virus replication when, following microscopic quality control, at least at one concentration of compound, no CPE nor any adverse effect is observed (image resembling untreated, uninfected cells). Multiple, independent experiments were performed. Chloroquine was used as positive control (CC_50_ = 89 ± 28 µM; EC_50_ = 10 ± 5 µM (SI = 8.9).

## 4. Conclusions

The chemical investigation of *Trigonostemon cherrieri* leaves EtOAc extract has led to the isolation in trace quantities of two new DDOs, named trigocherrierin A (**1**) and trigocherriolide E (**2**), and six chlorinated analogues, previously isolated from the bark and wood. Within this chemical series, trigocherrierin A (**1**) exhibited the most potent anti-CHIKV activity. Finally, from these data, it can be deduced that the chlorine atom is not essential for the biological activity.

## Supplementary Materials

Supplementary materials can be accessed at: http://www.mdpi.com/1420-3049/19/3/3617/s1.
