# Supplementary material for: Trigocherrierin A, a Potent Inhibitor of Chikungunya Virus Replication
Source: Molecules. 2014 Mar 24;19(3):3617–27. doi: 10.3390/molecules19033617 (PMC6270878; doi:10.3390/molecules19033617)
Supplement: Supplementary file 1 [file molecules-19-03617-s001.pdf]

Supporting information

Trigocherrierin A, a Potent Inhibitor of Chikungunya Virus Replication

Mélanie Bourjot [1], Pieter Leyssen [2], Johan Neyts [2], Vincent Dumontet [3] and Marc Litaudon [3,]*

[1] EA4267 Epithelial Functions and Dysfunctions, UFR of Medical and Pharmaceutical Sciences, 19 rue Ambroise Paré, 25030 Besançon, France; E-Mail: melanie.bourjot@univ-fcomte.fr

[2] Rega Institute for Medical Research (KU Leuven), Minderbroedersstraat 10, B3000, Leuven, Belgium; E-Mails: Pieter.Leyssen@rega.kuleuven.be (P.L.); Johan.Neyts@rega.kuleuven.be (J.N.)

[3] Gif Research Center, Institute of Chemistry of Natural Substances (ICSN), CNRS, Labex CEBA, 1, avenue de la Terrasse, 91198 Gif sur Yvette Cedex, France;
E-Mail: vincent.dumontet@cnrs.fr

***** Author to whom correspondence should be addressed; E-Mail: marc.litaudon@cnrs.fr;
Tel.: +33-169-823-085; Fax: +33-169-077-247.

Spectra of trigocherrierin A (**1**)

Figure S1 – ^{1}H-NMR spectrum ($CDCl_3$, 600 MHz) of trigocherrierin A (**1**)

Figure S2 – ^{13}C-NMR spectrum ($CDCl_3$, 150 MHz) of trigocherrierin A (**1**)

Figure S3 – HSQC spectrum ($CDCl_3$, 600 MHz) of trigocherrierin A (**1**)

Figure S4 – COSY spectrum ($CDCl_3$, 600 MHz) of trigocherrierin A (**1**)

Figure S5 – HMBC spectrum ($CDCl_3$, 600 MHz) of trigocherrierin A (**1**)

Figure S6 – ROESY spectrum ($CDCl_3$, 600 MHz) of trigocherrierin A (**1**)

Figure S7 – HR-ESIMS spectrum of trigocherrierin A (**1)**

Spectra of trigocherriolide E (**2**)

Figure S8 – ^{1}H-NMR spectrum ($CDCl_3$, 600 MHz) of trigocherriolide E (**2**)

Figure S9 – ^{13}C-NMR spectrum ($CDCl_3$, 150 MHz) of trigocherriolide E (**2**)

Figure S10 – HSQC spectrum ($CDCl_3$, 600 MHz) of trigocherriolide E (**2**)

Figure S11 – COSY spectrum ($CDCl_3$, 600 MHz) of trigocherriolide E (**2**)

Figure S12 – HMBC spectrum ($CDCl_3$, 600 MHz) of trigocherriolide E (**2**)

Figure S13 – ROESY spectrum ($CDCl_3$, 600 MHz) of trigocherriolide E (**2**)

Figure S14 – HR-ESIMS spectrum of trigocherriolide E (**2**)

Figure S1. ^{1}H-NMR spectrum ($CDCl_3$, 600 MHz) of trigocherrierin A (**1**).

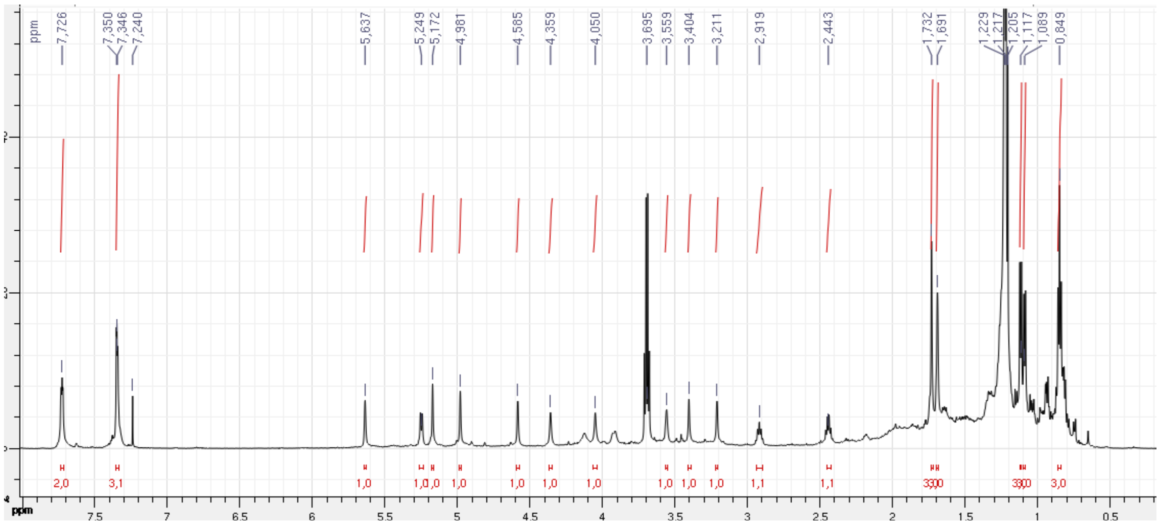

Figure S2. ^{13}C-NMR spectrum ($CDCl_3$, 150 MHz) of trigocherrierin A (**1**).

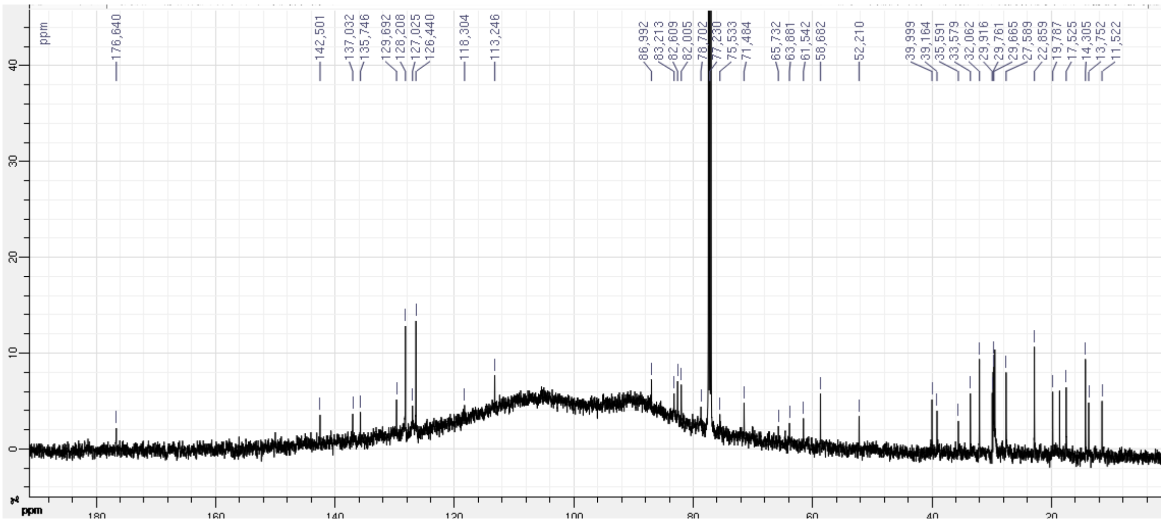

Figure S3. HSQC spectrum ($CDCl_3$, 600 MHz) of trigocherrierin A (**1**).

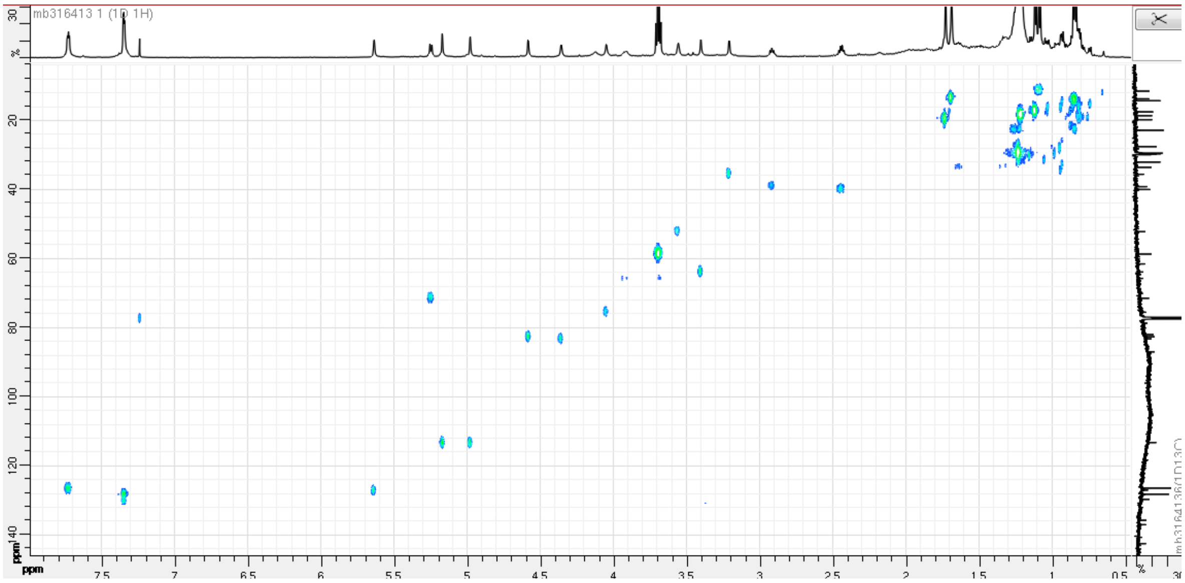

Figure S4. COSY spectrum ($CDCl_3$, 600 MHz) of trigocherrierin A (**1**).

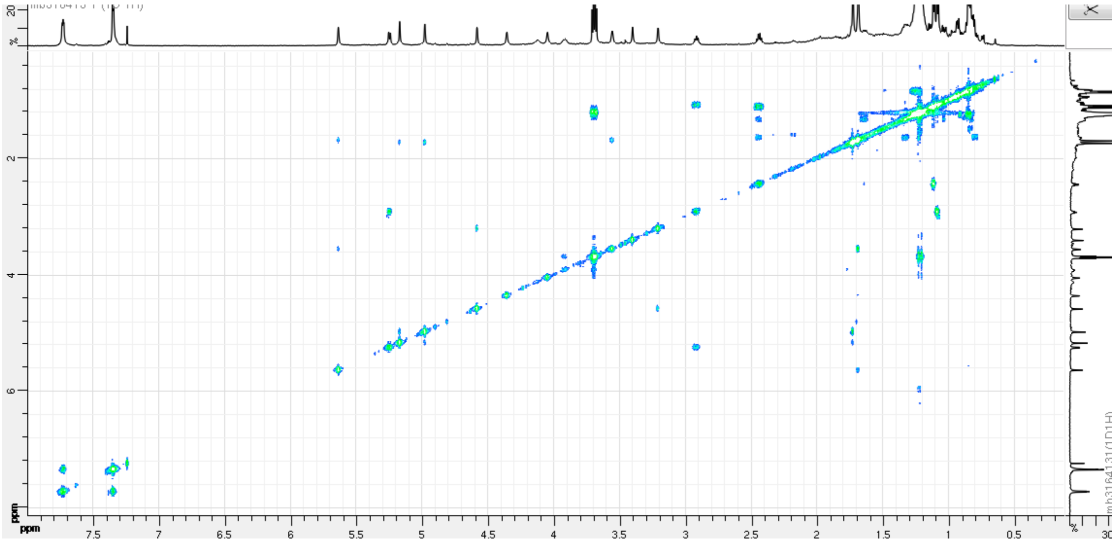

Figure S5. HMBC spectrum ($CDCl_3$, 600 MHz) of trigocherrierin A (**1**).

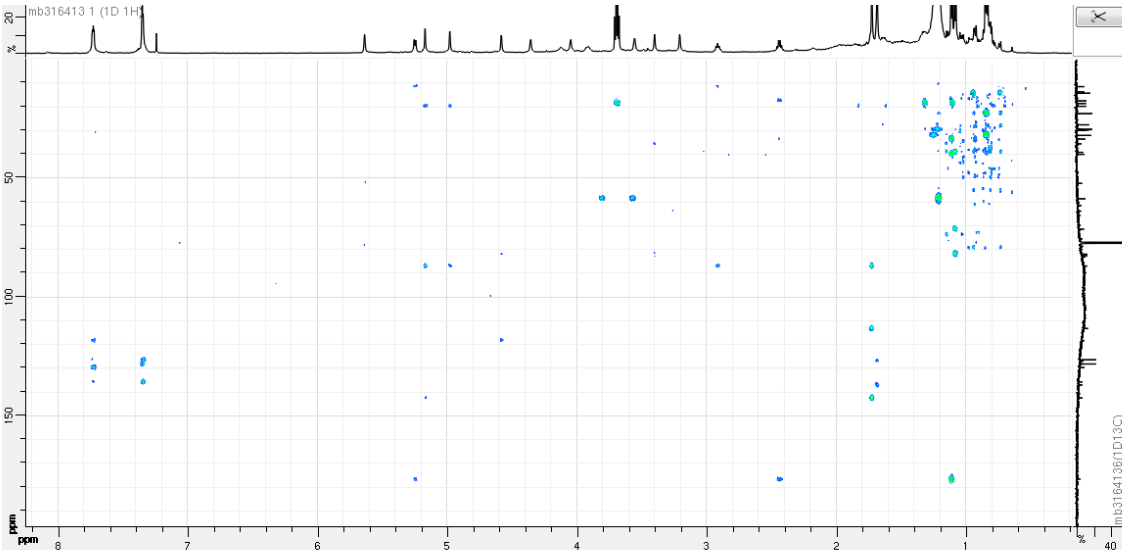

Figure S6. ROESY spectrum ($CDCl_3$, 600 MHz) of trigocherrierin A (**1**).

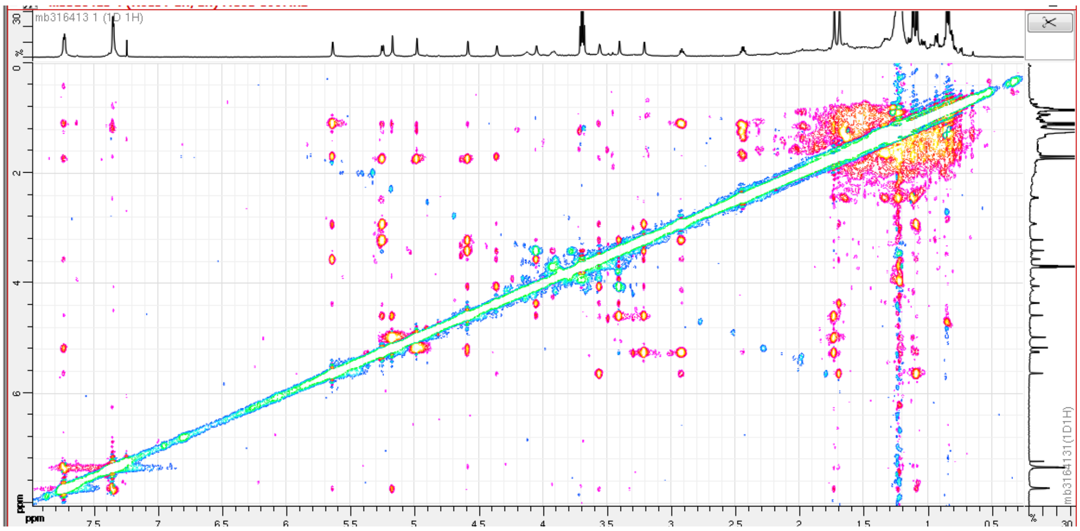

Figure S7. HR-ESIMS spectrum of compound **1** (M+H)$^+$.

Elemental Composition Report Page 1

Single Mass Analysis
Tolerance = 20.0 PPM / DBE: min = -1.5, max = 50.0
Element prediction: Off
Number of isotope peaks used for i-FIT = 9

HR-ESIMS (M+H)+ of Trigocherrierin A

Monoisotopic Mass, Even Electron Ions
86 formula(e) evaluated with 4 results within limits (all results (up to 1000) for each mass)
Elements Used:
C: 1-100 H: 0-100 O: 0-10
31-Jan-2012 2::2::2 MeOH+CH2Cl2 LCT Premier XE KE483
GUE_MB3-164-13 29 (0.752) Cm (29:38) 1: TOF MS ES+ 8.58e+003

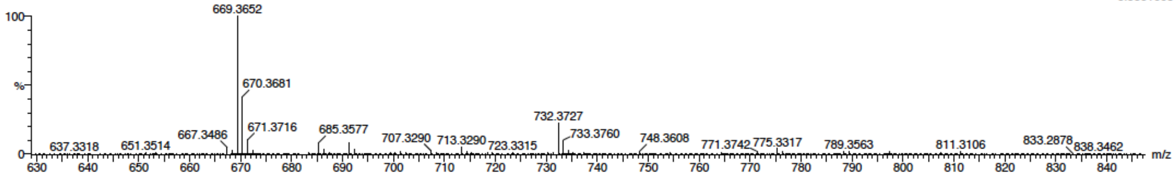

Mass	Calc. Mass	mDa	PPM	DBE	i-FIT	i-FIT (Norm)	Formula
Minimum:				-1.5			
Maximum:		5.0	20.0	50.0			
669.3652	669.3639	1.3	1.9	12.5	184.5	0.0	C38 H53 O10
	669.3580	7.2	10.8	21.5	194.6	10.0	C45 H49 O5
	669.3733	-8.1	-12.1	25.5	198.2	13.6	C49 H49 O2
	669.3521	13.1	19.6	30.5	202.0	17.4	C52 H45

Figure S8. ^{1}H-NMR spectrum ($CDCl_3$, 600 MHz) of trigocherriolide E (**2**).

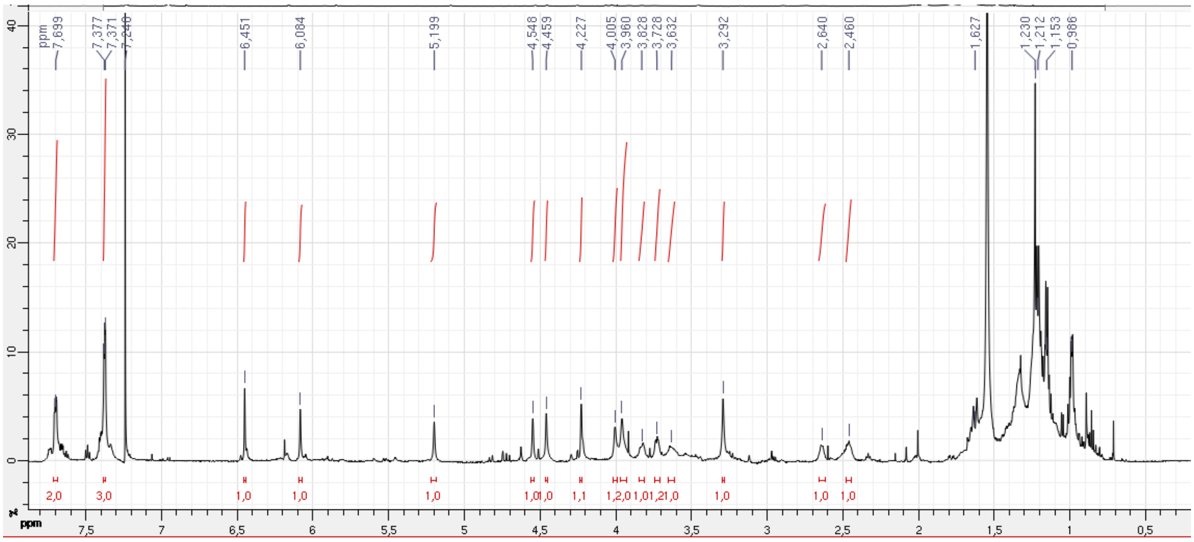

Figure S9. ^{13}C-NMR spectrum ($CDCl_3$, 150 MHz) of trigocherriolide E (**2**).

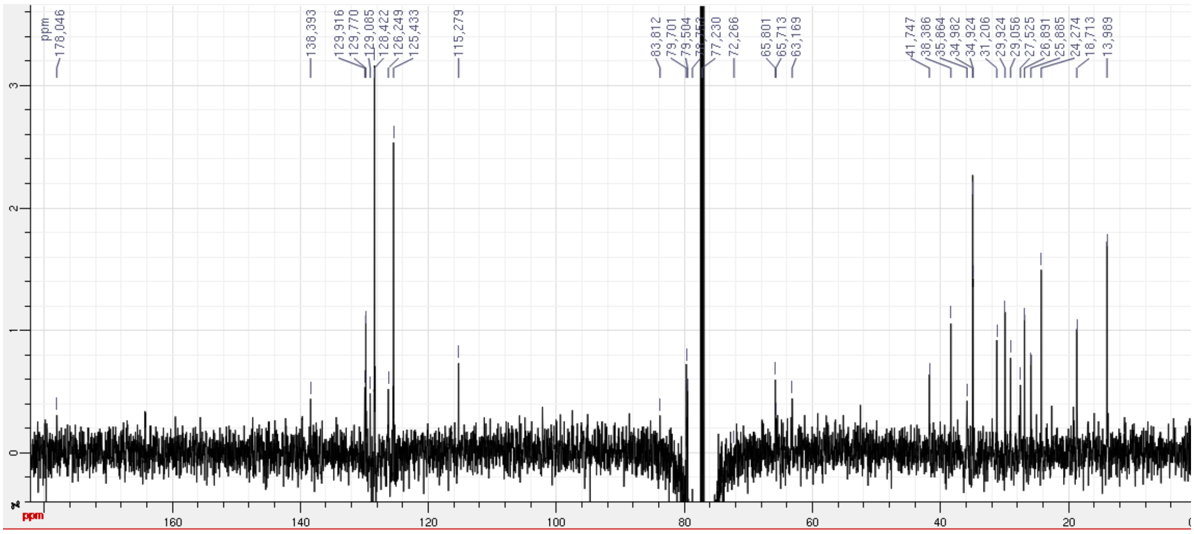

Figure S10. HSQC spectrum ($CDCl_3$, 600 MHz) of trigocherriolide E (**2**).

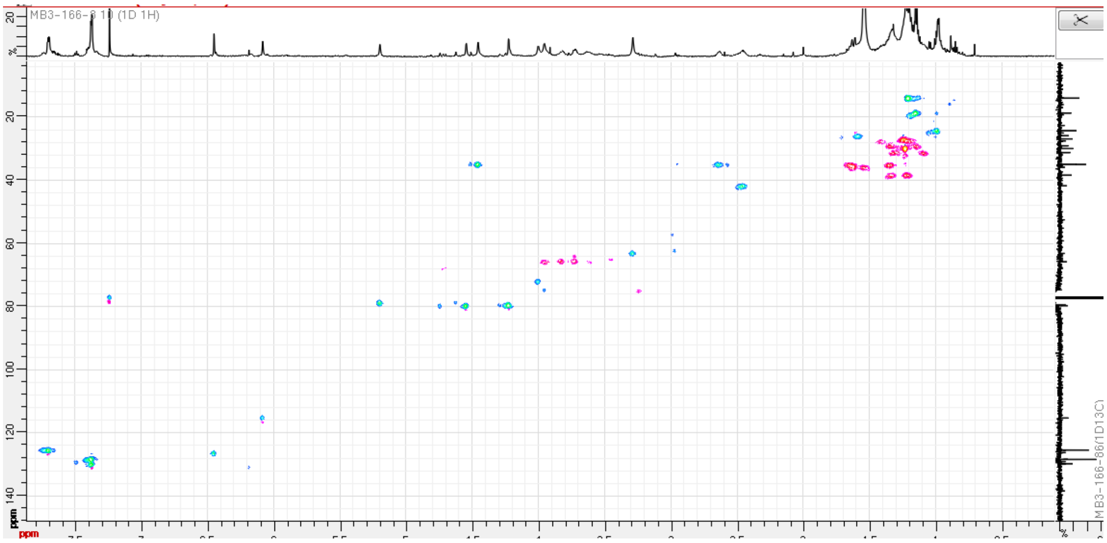

Figure S11. COSY spectrum ($CDCl_3$, 600 MHz) of trigocherriolide E (**2**).

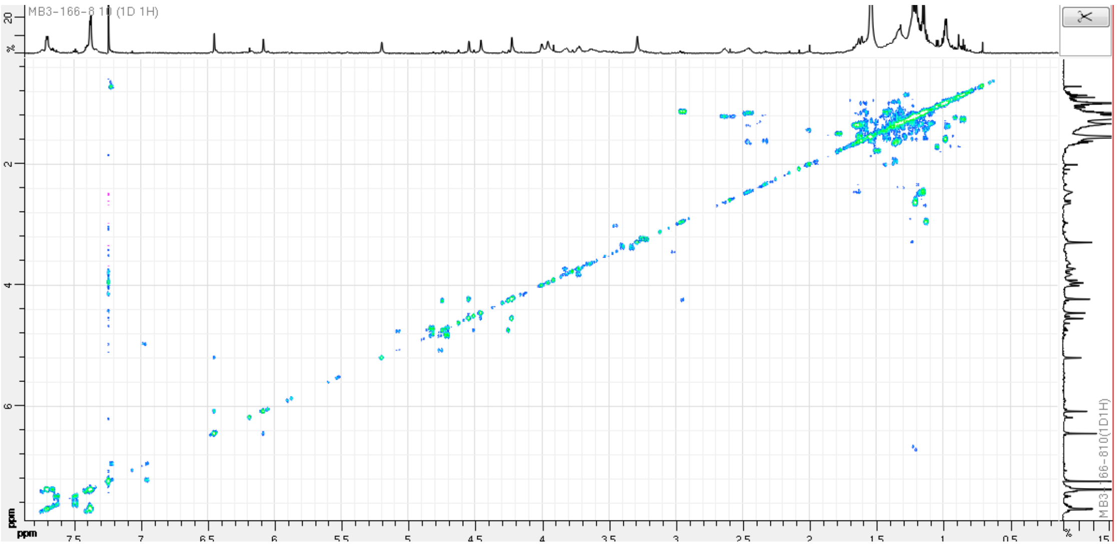

Figure S12. HMBC spectrum ($CDCl_3$, 600 MHz) of trigocherriolide E (**2**).

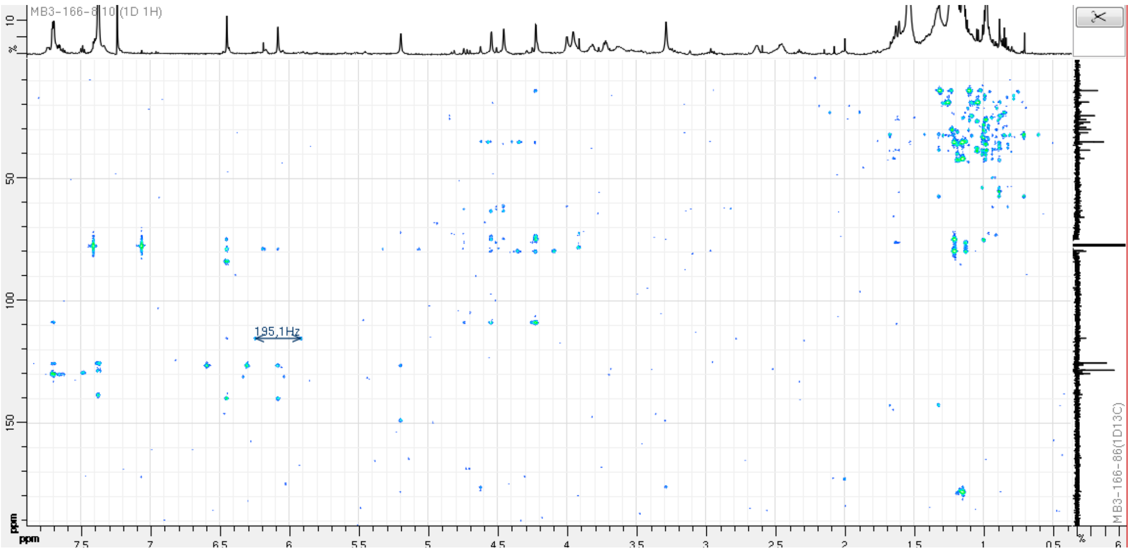

Figure S13. ROESY spectrum ($CDCl_3$, 600 MHz) of trigocherriolide E (**2**).

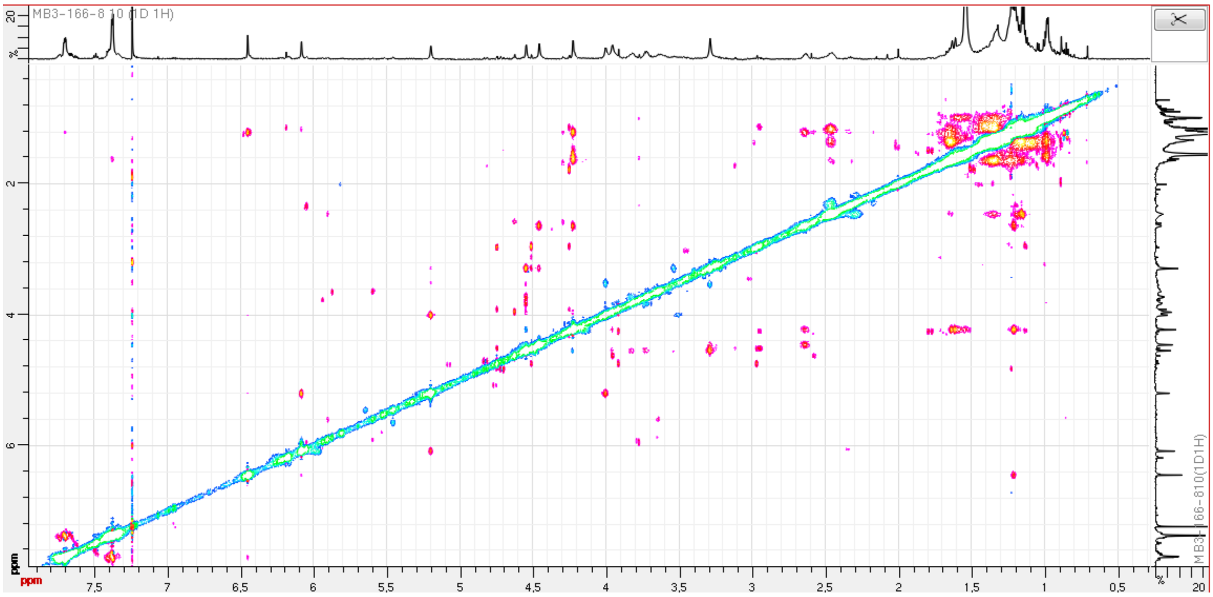

Figure S14. HR-ESIMS spectrum of trigocherriolide E (**2**).

Elemental Composition Report **Page 1**

Single Mass Analysis
Tolerance = 5.0 PPM / DBE: min = -1.5, max = 50.0
Element prediction: Off
Number of isotope peaks used for i-FIT = 9

HR-ESIMS (M+H)+ of Trigocheriolide E

Monoisotopic Mass, Even Electron Ions
24 formula(e) evaluated with 1 results within limits (all results (up to 1000) for each mass)
Elements Used:
C: 0-40 H: 10-120 O: 0-12 Cl: 1-1
27-Feb-2012 10:34:22
GUE_MB3-166-8 23 (0.607) Cm (17:35)

MeOH+CH2Cl2

LCT Premier XE KE483
1: TOF MS ES+
1.41e+004

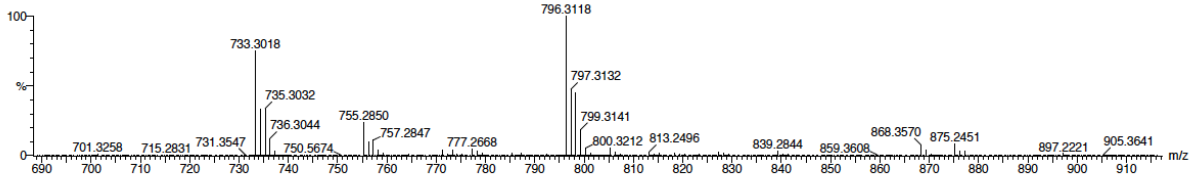

Minimum:				-1.5			
Maximum:		5.0	5.0	50.0			
Mass	Calc. Mass	mDa	PPM	DBE	i-FIT	i-FIT (Norm)	Formula
733.3018	733.2991	2.7	3.7	13.5	307.4	0.0	C38 H50 O12 Cl
